# Antimicrobial resistance profiles of *Mammaliicoccus sciuri* and *Staphylococcus* spp. isolated from reptiles undergoing rehabilitation in Northeastern Brazil

**DOI:** 10.1007/s42770-026-01909-9

**Published:** 2026-04-27

**Authors:** Denny Parente de Sá Barreto Maia Leite, Eduarda Beatriz Rodrigues Barbosa, Victor Oliveira Herculano de Farias, Valdir Vieira da Silva, Lucilene Martins Trindade Gonçalves, Pollyanne Raysa Fernandes de Oliveira, Rafaela Silva Santos, Maria Eduarda Uchôa Cavalcanti Moreira da Silva, Gustavo de Oliveira Alves Pinto, José Givanildo da Silva, Karolina Rosa Fernandes Beraldo, Maria Aparecida Juliano, Rinaldo Aparecido Mota

**Affiliations:** 1 Department of Veterinary Medicine, Federal Rural University of Pernambuco, Recife, Pernambuco Brazil; 2 Department of Preventive Veterinary Medicine and Animal Production, School of Veterinary Medicine and Animal Science, Federal University of Bahia, Salvador, Bahia Brazil; 3 Department of Biophysics, Paulista School of Medicine - Federal University of São Paulo, São Paulo, São Paulo Brazil

**Keywords:** Microbiological surveillance, Multidrug resistance, One Health, Wildlife.

## Abstract

Antimicrobial resistance (AMR) in wildlife-associated bacteria represents a growing One Health concern. This study described the occurrence and antimicrobial susceptibility of *Mammaliicoccus sciuri* and *Staphylococcus* spp. isolated from reptiles undergoing rehabilitation in northeastern Brazil. Oropharyngeal and cloacal samples from 87 reptiles yielded 87 isolates identified by MALDI-TOF MS, and antimicrobial susceptibility was assessed by disk diffusion. Five bacterial species were detected, with a predominance of *M. sciuri* (79/87; 90.8%). Resistance to oxacillin was the most frequent finding (40/87; 46.0%), followed by erythromycin (16/87; 18.4%), while resistance to other antimicrobials remained low. In total, 46/87 (52.9%) isolates were resistant to at least one antimicrobial and 12/87 (13.8%) were classified as multidrug-resistant, mainly from bacteria isolated from *Iguana iguana*, *Chelonoidis carbonaria*, *Boa constrictor*, and *Tupinambis teguixin*. MAR indices ranged from 0.00 to 0.86, with values > 0.20 in 25.3% of isolates. These findings indicate that rehabilitating reptiles may act as reservoirs of oxacillin-resistant staphylococci, reinforcing the need to include wildlife rehabilitation centers in AMR surveillance strategies.

**Graphical Abstract:**

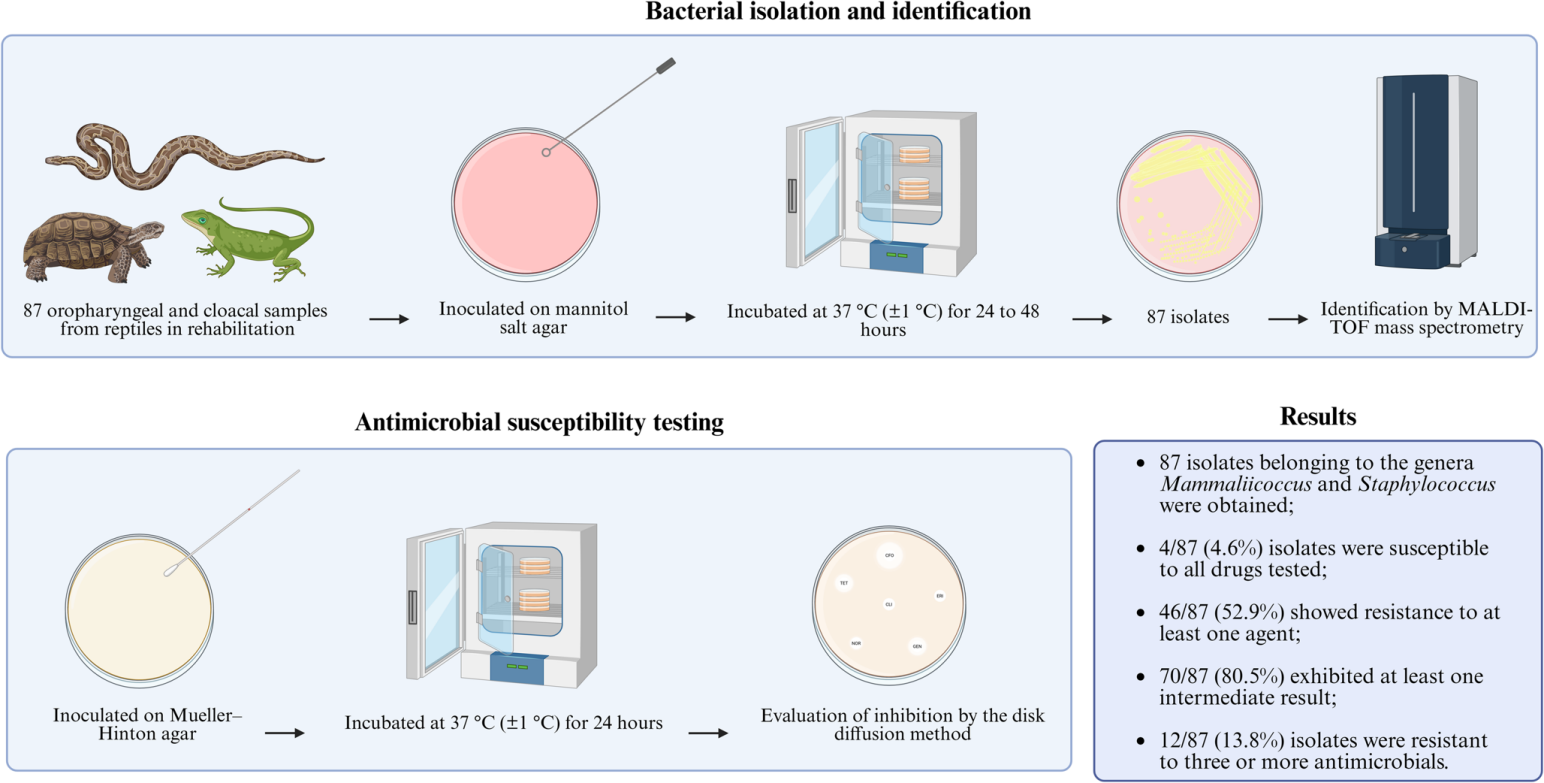

**Supplementary Information:**

The online version contains supplementary material available at 10.1007/s42770-026-01909-9.

## Introduction

Antimicrobial resistance (AMR) is a natural phenomenon that has been intensified by selective pressures resulting from the inappropriate use of antibiotics in human, veterinary, and agricultural medicine, constituting a typical challenge within the One Health framework [1–3]. At the interface among humans, the environment, and wildlife, this condition compromises therapeutic efficacy and increases sanitary risks [3].

Among the microorganisms of interest, *Staphylococcus* spp. are recognized as common colonizers of the skin and mucous membranes, exhibiting opportunistic behavior and broad ecological plasticity [4, 5]. *Mammaliicoccus sciuri* is also noteworthy, as this species was recently reclassified and is still frequently reported in the literature as *Staphylococcus sciuri* [6, 7]. These bacteria may harbor resistance determinants to multiple classes of antibiotics, such as β-lactams, fluoroquinolones, aminoglycosides, and tetracyclines, thereby exacerbating the AMR problem [8].

During the management and rehabilitation of wild animals, close contact with humans, other animals, and contaminated surfaces favors the dissemination of multidrug-resistant strains [9, 10]. In reptiles, these species have frequently been associated with resistance profiles and biofilm production in captivity [11], as well as with the colonization of chelonians admitted to official wildlife centers in Brazil [12]. In this context, the aim of this study was to isolate, identify, and determine the antimicrobial susceptibility profile of *Staphylococcus* spp. and *M. sciuri* recovered from oropharyngeal and cloacal samples of reptiles admitted to the Wildlife Screening and Rehabilitation Center of Pernambuco (CETRAS–Tangará), with emphasis on the detection of multidrug resistance.

## Materials and methods

### Ethical approval

This project was approved by the Ethics Committee on the Use of Animals of the Federal Rural University of Pernambuco (CEUA-UFRPE, protocol no. 4094160421), by the Brazilian Authorization and Information System for Biodiversity (SISBIO license no. 82873), and by the National System for the Management of Genetic Heritage and Associated Traditional Knowledge (SisGen license no. AC42592).

### Study site and sampled population

The study was conducted at the Tangara Wildlife Screening and Rehabilitation Center (CETRAS Tangara), located in Recife, Pernambuco, northeastern Brazil (7°56′47″S, 34°58′47″W), within the Atlantic Forest biome. Oropharyngeal and cloacal samples were collected from 87 reptiles belonging to the orders Squamata and Testudines during routine handling procedures conducted by CETRAS staff (Table 1).

**Table 1:** List of reptiles sampled according to order, family, and species

Order	Family	Popular name	Scientific name	No. of animals
Squamata	*Teiidae*	Tegu lizard	*Tupinambis teguixin*	**1**
	*Iguanidae*	Green iguana	*Iguana iguana*	**8**
	*Boidae*	Boa constrictor	*Boa constrictor*	**5**
	*Dipsadidae*	Mussurana	*Clelia clelia*	**1**
Testudines	*Cheloniidae*	Geoffroy’s side-necked turtles	*Phrynops geoffroanus*	**3**
	*Testudinidae*	Red-footed tortoises	*Chelonoidis carbonaria*	**69**

### Bacterial isolation and identification

Bacterial isolation was performed by inoculating the samples onto Brain Heart Infusion (BHI) agar and Mannitol Salt Agar (MSA). The inoculated plates were incubated at 37 °C (± 1 °C) and inspected after 24 h. All plates were subsequently maintained under the same conditions and re-evaluated at 48 h to detect late bacterial growth [13]. After incubation, the plates were examined for bacterial growth, and presumptive bacterial colonies were subjected to Gram staining and catalase testing [14]. Colonies suggestive of *Staphylococcus* spp. and *Mammaliicoccus* spp. were subcultured onto fresh MSA plates and identified by matrix-assisted laser desorption/ionization time-of-flight mass spectrometry (MALDI-TOF MS) according to Clark et al. [15].

### Antimicrobial susceptibility testing

The phenotypic resistance of bacterial isolates to different classes of antimicrobials was evaluated using the disk diffusion method. The tests were performed on Mueller–Hinton agar plates with bacterial suspensions adjusted to 0.5 on the McFarland turbidity scale, following CLSI and EUCAST guidelines. The reference strains *Staphylococcus aureus* (ATCC 25923) and *Escherichia coli* (ATCC 25922) were used as positive controls [16, 17].

The antibiotic disks used included cefoxitin (CFO, 30 µg), clindamycin (CLI, 2 µg), erythromycin (ERI, 15 µg), gentamicin (GEN, 10 µg), norfloxacin (NOR, 10 µg), oxacillin (OXA, 1 µg), and tetracycline (TET, 30 µg) to assess the resistance profile of the isolates. Interpretation of the inhibition zone diameters was based on CLSI VET01S, 7th edition [17], and EUCAST [18] guidelines. For *M. sciuri* isolates, due to the absence of species-specific clinical breakpoints, interpretive criteria for coagulase-negative staphylococci were applied [19].

### Statistical analysis

The classification of isolates as multidrug-resistant (MDR) followed the definitions proposed by Magiorakos et al. [20] and Sweeney et al. [21] and was based on non-susceptibility to at least one agent in three or more antimicrobial classes. The multiple antibiotic resistance (MAR) index was calculated according to Krumperman [22] using the formula a/b, where ‘a’ represents the number of antibiotics to which an isolate was resistant and ‘b’ represents the total number tested. Data were summarized using descriptive statistics. Absolute and relative frequencies were calculated for bacterial isolation by host species and anatomical sampling site, and antimicrobial susceptibility results were expressed as proportions of susceptible, intermediate, and resistant isolates. The mean MAR index was calculated across isolates. All analyses and graphs were performed using GraphPad Prism 10.

## Results

A total of 87 isolates belonging to the genera *Mammaliicoccus* and *Staphylococcus* were obtained, with most recovered from *Chelonoidis carbonaria*, followed by *Iguana iguana*, *Boa constrictor*, *Phrynops geoffroanus*, and *Clelia clelia*. The results of the bacterial cultures according to reptile species are presented in Table 2.

**Table 2:** Bacteriological culture results from reptile samples by host species

Species	Sampled animals	Positive bacteriological cultures	Bacterial isolates
*Tupinambis teguixin*	1/87 (1,1%)	1/1 (100%)	2/87 (2,3%)
*Iguana iguana*	8/87 (9,2%)	7/8 (87,5%)	10/87 (11,5%)
*Boa constrictor*	5/87 (5,7%)	4/5 (80,0%)	11/87 (12,6%)
*Clelia clelia*	1/87 (1,1%)	1/1 (100%)	2/87 (2,3%)
*Phrynops geoffroanus*	3/87 (3,4%)	2/3 (66,7%)	4/87 (4,6%)
*Chelonoidis carbonaria*	69/87 (79,3%)	32/69 (46,4%)	58/87 (66,7%)

Five bacterial species were identified. *M. sciuri* was by far the predominant species, accounting for 79/87 (90.8%). A slightly higher recovery rate was observed from cloacal samples (49/87; 56.3%) compared with oropharyngeal swabs (38/87; 43.7%), and the distribution of isolates according to host species, bacterial species, and anatomical sampling site is shown in Table  3.

**Table 3:** Distribution of bacterial species by reptile host species and anatomical sampling site

Host species	Total isolates in host (*n*)	Bacterial species	Cloaca *n* (%)	Oropharynx *n* (%)	Total in host *n* (%)
*Tupinambis teguixin*	2	*Mammaliicoccus sciuri*	0 (0.0)	2 (100.0)	2 (100.0)
*Iguana iguana*	10	*Mammaliicoccus sciuri*	6 (60.0)	4 (40.0)	10 (100.0)
*Chelonoidis carbonaria*	58	*Mammaliicoccus sciuri*	33 (62.3)	20 (37.7)	53 (91.4)
*Chelonoidis carbonaria*	58	*Staphylococcus saprophyticus*	2 (100.0)	0 (0.0)	2 (3.4)
*Chelonoidis carbonaria*	58	*Staphylococcus xylosus*	0 (0.0)	3 (100.0)	3 (5.2)
*Boa constrictor*	11	*Mammaliicoccus sciuri*	4 (44.4)	5 (55.6)	9 (81.8)
*Boa constrictor*	11	*Staphylococcus xylosus*	1 (100.0)	0 (0.0)	1 (9.1)
*Boa constrictor*	11	*Staphylococcus aureus*	1 (100.0)	0 (0.0)	1 (9.1)
*Phrynops geoffroanus*	4	*Mammaliicoccus sciuri*	2 (66.7)	1 (33.3)	3 (75.0)
*Phrynops geoffroanus*	4	*Staphylococcus gallinarum*	0 (0.0)	1 (100.0)	1 (25.0)
*Clelia clelia*	2	*Mammaliicoccus sciuri*	0 (0.0)	2 (100.0)	2 (100.0)

**Fig. 1:**
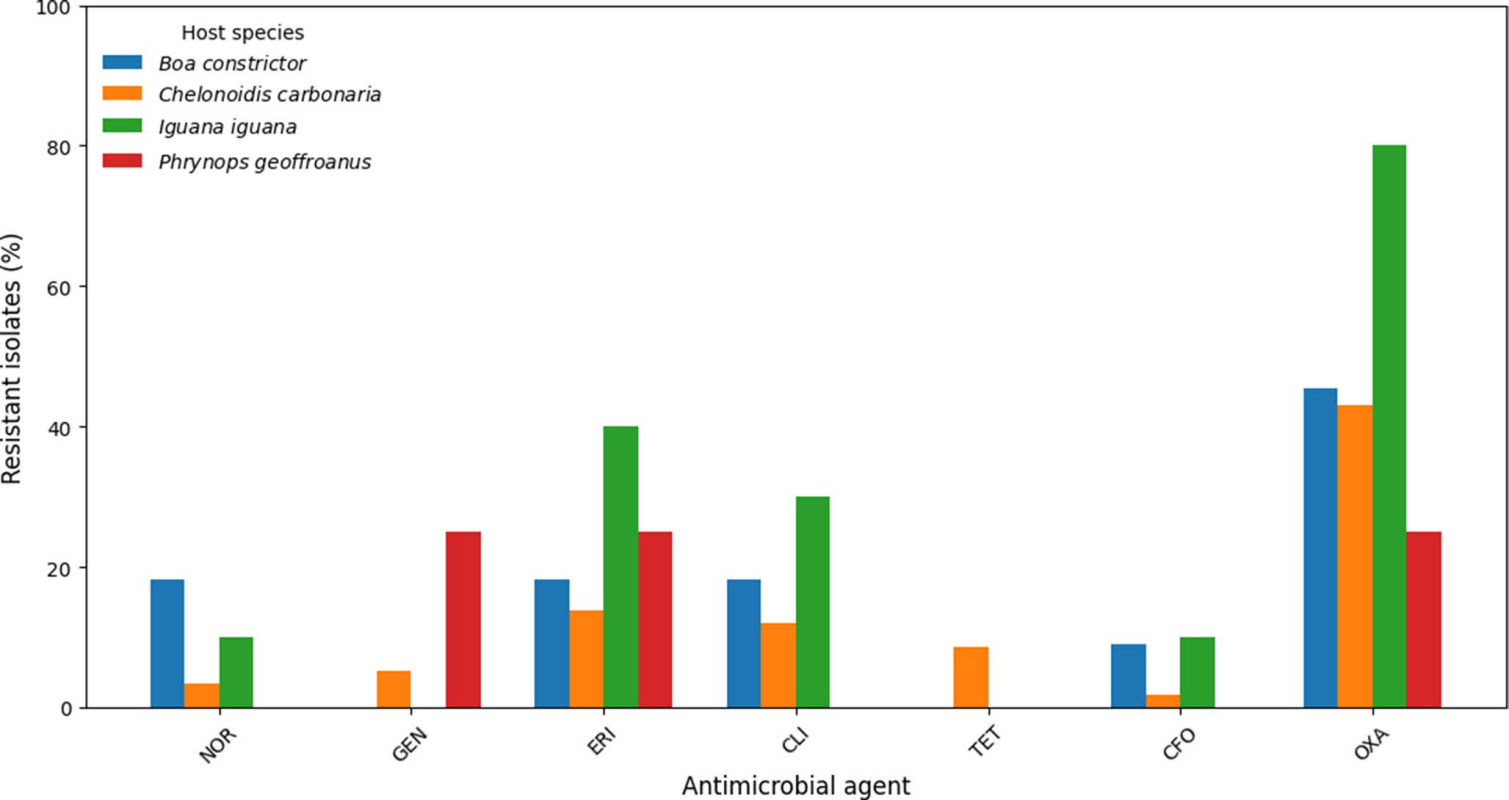
Proportion of resistant isolates according to host species and antimicrobial agent

Overall antimicrobial susceptibility analysis showed that most isolates remained susceptible to the antimicrobials tested, whereas resistance to oxacillin was the most frequent finding (40/87; 46.0%). Detailed susceptibility profiles by bacterial species are presented in Table 4. Considering all antimicrobials, 46/87 (52.9%) isolates were resistant to at least one agent and 12/87 (13.8%) were classified as multidrug-resistant. The distribution of resistant isolates according to host species is shown in Table 5, and resistance patterns by antimicrobial class are illustrated in Fig. 1.

**Table 4:** Antimicrobial resistance profiles of *Mammaliicoccus sciuri* and *Staphylococcus* spp. isolates recovered from reptiles, stratified by bacterial species

Bacterial species	Antimicrobial	Sensitive	Intermediate	Resistant
*Mammaliicoccus sciuri*	Norfloxacin	65/79 (82,3%)	9/79 (11,4%)	5/79 (6,3%)
	Gentamicin	67/79 (84,8%)	6/79 (7,6%)	6/79 (7,6%)
	Erythromycin	57/79 (72,2%)	6/79 (7,6%)	16/79 (20,3%)
	Clindamycin	31/79 (39,2%)	34/79 (43,0%)	14/79 (17,7%)
	Tetracycline	65/79 (82,3%)	8/79 (10,1%)	6/79 (7,6%)
	Cefoxitin	65/79 (82,3%)	10/79 (12,7%)	4/79 (5,1%)
	Oxacillin	14/79 (17,7%)	27/79 (34,2%)	38/79 (48,1%)
*Staphylococcus xylosus*	Norfloxacin	4/4 (100,0%)	0/4 (0,0%)	0/4 (0,0%)
	Gentamicin	4/4 (100,0%)	0/4 (0,0%)	0/4 (0,0%)
	Erythromycin	4/4 (100,0%)	0/4 (0,0%)	0/4 (0,0%)
	Clindamycin	2/4 (50,0%)	2/4 (50,0%)	0/4 (0,0%)
	Tetracycline	4/4 (100,0%)	0/4 (0,0%)	0/4 (0,0%)
	Cefoxitin	4/4 (100,0%)	0/4 (0,0%)	0/4 (0,0%)
	Oxacillin	3/4 (75,0%)	0/4 (0,0%)	1/4 (25,0%)
*Staphylococcus saprophyticus*	Norfloxacin	2/2 (100,0%)	0/2 (0,0%)	0/2 (0,0%)
	Gentamicin	2/2 (100,0%)	0/2 (0,0%)	0/2 (0,0%)
	Erythromycin	2/2 (100,0%)	0/2 (0,0%)	0/2 (0,0%)
	Clindamycin	1/2 (50,0%)	1/2 (50,0%)	0/2 (0,0%)
	Tetracycline	2/2 (100,0%)	0/2 (0,0%)	0/2 (0,0%)
	Cefoxitin	2/2 (100,0%)	0/2 (0,0%)	0/2 (0,0%)
	Oxacillin	0/2 (0,0%)	2/2 (100,0%)	0/2 (0,0%)
*Staphylococcus gallinarum*	Norfloxacin	1/1 (100,0%)	0/1 (0,0%)	0/1 (0,0%)
	Gentamicin	1/1 (100,0%)	0/1 (0,0%)	0/1 (0,0%)
	Erythromycin	1/1 (100,0%)	0/1 (0,0%)	0/1 (0,0%)
	Clindamycin	1/1 (100,0%)	0/1 (0,0%)	0/1 (0,0%)
	Tetracycline	1/1 (100,0%)	0/1 (0,0%)	0/1 (0,0%)
	Cefoxitin	1/1 (100,0%)	0/1 (0,0%)	0/1 (0,0%)
	Oxacillin	0/1 (0,0%)	1/1 (100,0%)	0/1 (0,0%)
*Staphylococcus aureus*	Norfloxacin	0/1 (0,0%)	1/1 (100,0%)	0/1 (0,0%)
	Gentamicin	1/1 (100,0%)	0/1 (0,0%)	0/1 (0,0%)
	Erythromycin	1/1 (100,0%)	0/1 (0,0%)	0/1 (0,0%)
	Clindamycin	0/1 (0,0%)	1/1 (100,0%)	0/1 (0,0%)
	Tetracycline	1/1 (100,0%)	0/1 (0,0%)	0/1 (0,0%)
	Cefoxitin	1/1 (100,0%)	0/1 (0,0%)	0/1 (0,0%)
	Oxacillin	0/1 (0,0%)	0/1 (0,0%)	1/1 (100,0%)

**Table 5:** Antimicrobial resistance profiles of bacterial isolates according to reptile host species

Host species	No. of isolates	Resistence to ≥ 1 antimicrobial	Resistance to ≥ 3 antimicrobials *n* (%)
*Iguana iguana*	10	9/10 (90,0%)	3/10 (30,0%)
*Chelonoidis carbonaria*	58	26/58 (44,8%)	7/58 (12,1%)
*Boa constrictor*	11	7/11 (63,6%)	1/11 (9,1%)
*Phrynops geoffroanus*	4	2/4 (50,0%)	0/4 (0,0%)
*Tupinambis teguixin*	2	1/2 (50,0%)	1/2 (50,0%)
*Clelia clelia*	2	1/2 (50,0%)	0/2 (0,0%)

The number of antimicrobials to which each isolate exhibited resistance ranged from 0/7 to 6/7, with MAR index values varying between 0.00 and 0.86 (mean: 0.15). A total of 22/87 isolates (25.3%) showed MAR values > 0.20. Among the host species, MDR isolates were identified in *I. iguana* (3/10; 30.0%), *T. teguixin* (1/2; 50.0%), *B. constrictor* (1/11; 9.1%), and *C. carbonaria* (7/58; 12.1%), whereas *C. clelia* and *P. geoffroanus* did not present multidrug-resistant isolates.

## Discussion

This study demonstrated a higher prevalence of *M. sciuri* among isolates obtained from rehabilitated reptiles, and the high frequency observed corroborates previous reports indicating that species of this genus, as well as coagulase-negative staphylococci (CoNS), are common in reptiles and may act both as commensal organisms and as potential reservoirs of antimicrobial resistance [12, 23].

In red-footed tortoises maintained in Minas Gerais, for example, *S. sciuri* (currently *M. sciuri*) accounted for 81.3% of isolates, and *S. xylosus* for 12.5%, a profile like that observed in *C. carbonaria* in our study [12]. Likewise, analyses of captive reptiles in Europe also confirmed the distribution of *M. sciuri* and *S. xylosus* [8]. These findings suggest that these bacteria play a role as habitual members of the skin and cloacal microbiota of reptiles.

Regarding bacterial diversity, the detection of *S.aureus*, *S. gallinarum*, and *S. saprophyticus* at low frequencies expands the list of species previously reported in reptiles, albeit sporadically. Cristina et al. [24] identified *S. aureus* in companion animals in Europe, although with lower prevalence compared to other environmental and opportunistic bacteria. The presence of *S. aureus* in reptiles, even if minor, is noteworthy due to its recognized zoonotic potential and the emergence of resistant lineages [25].

Regarding the resistance profile, *M. sciuri* isolates exhibited considerable rates of resistance to oxacillin (48.1%) and lower rates to erythromycin (20.3%), clindamycin (17.7%), and tetracycline (7.6%). These findings are consistent with those reported in turtles by Santana et al. [12], who observed resistance of 35.5% to penicillin and 29.1% to tetracycline, but no resistance to oxacillin. In contrast, Strompfová et al. [8] reported near-universal resistance of reptile staphylococci to ampicillin (100%) and cefoxitin (98%), highlighting geographic and methodological variations that may explain differences in resistance rates.

The detection of oxacillin resistance is particularly relevant, as it supports the hypothesis of the circulation of β-lactam–resistant phenotypes in reptiles, even though the frequency of MDR (13.8%) was lower than that reported in exotic animals in the Iberian Peninsula (21%) [26].

Analysis by host species showed that iguanas and tegus were frequent carriers of MDR isolates, 30.0% and 50.0%, respectively. This uneven distribution among hosts parallels the findings of Cristina et al. [24], who reported variation in resistance occurrence among different groups of pet reptiles, with chelonians being the most susceptible to diseases associated with resistant bacteria. These differences suggest that ecological and physiological factors specific to each species may influence bacterial colonization and the risk of resistance dissemination [10, 11].

From a One Health perspective, the results of our study reinforce previous warnings regarding the importance of reptiles as potential reservoirs of resistant bacteria. A recent review highlights that resistance in staphylococci from wildlife is strongly influenced by anthropogenic pressures, such as contact with urban environments and domestic or hospital waste [25].

Moreover, Ebani [23] emphasizes that reptiles, often asymptomatic, may act as carriers of resistant bacteria, posing a risk of transmission to humans and other animals. These findings, together with the detection of oxacillin-resistant *S. aureus*, underscore the need for continuous surveillance, given that methicillin-resistant *Staphylococcus aureus* (MRSA) infections in exotic animals have already been reported [25].

Although studies such as that of Mlangeni et al. [27] have primarily focused on *Salmonella* spp. in reptiles from South Africa, the authors also highlighted the presence of antimicrobial resistance associated with zoonotic risk. This observation aligns with the findings reported here, in which the detection of multidrug-resistant isolates across different reptile species underscores the interface between animals, human, and environmental health. In the same direction, Muñoz-Ibarra et al. [26] demonstrated that exotic animals may harbor bacteria resistant to critically important antimicrobials for human use, further raising concern within the One Health framework.

This study has important limitations, including the small number of individuals from some host species, the restricted antimicrobial panel, and the absence of genotypic and molecular typing analyses. As a result, resistance was assessed only phenotypically, and the genetic relatedness among isolates could not be determined. Therefore, the findings should be interpreted as descriptive and exploratory. Even so, the study provides baseline data on antimicrobial resistance in bacteria isolated from wild reptiles undergoing rehabilitation in northeastern Brazil, an underexplored setting within the One Health framework, and it may inform future molecular epidemiological investigations.

## Conclusion

From a One Health perspective, these results provide preliminary evidence that reptiles admitted to rehabilitation and screening centers can harbor antimicrobial-resistant bacteria, including multidrug-resistant isolates. While exploratory, the findings support the inclusion of wildlife rehabilitation settings in broader antimicrobial resistance surveillance efforts and justify further studies incorporating molecular approaches and investigation of environmental sources of contamination.

## Supplementary Information

Below is the link to the electronic supplementary material.

Supplementary Material 1 (DOCX 46.2 KB)

Supplementary Material 2 (DOCX 249 KB)
