# Supplementary figures and images for: Antimicrobial resistance profiles of Mammaliicoccus sciuri and Staphylococcus spp. isolated from reptiles undergoing rehabilitation in Northeastern Brazil
Source: Braz J Microbiol. 2026 Apr 27;57(1):123. doi: 10.1007/s42770-026-01909-9 (PMC13121670; doi:10.1007/s42770-026-01909-9)

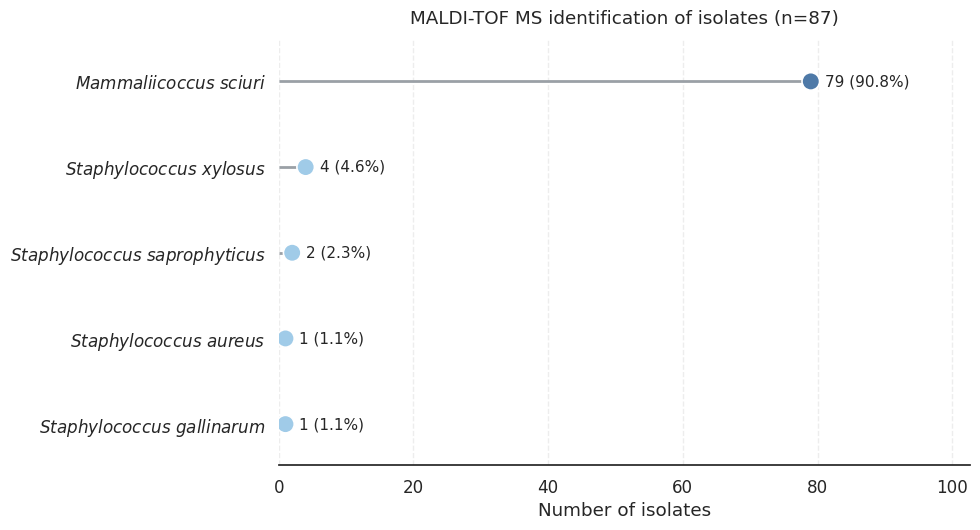

Supplement: Supplementary file 1 — Supplementary Material 1 (DOCX 46.2 KB) [file 42770_2026_1909_MOESM1_ESM.png]

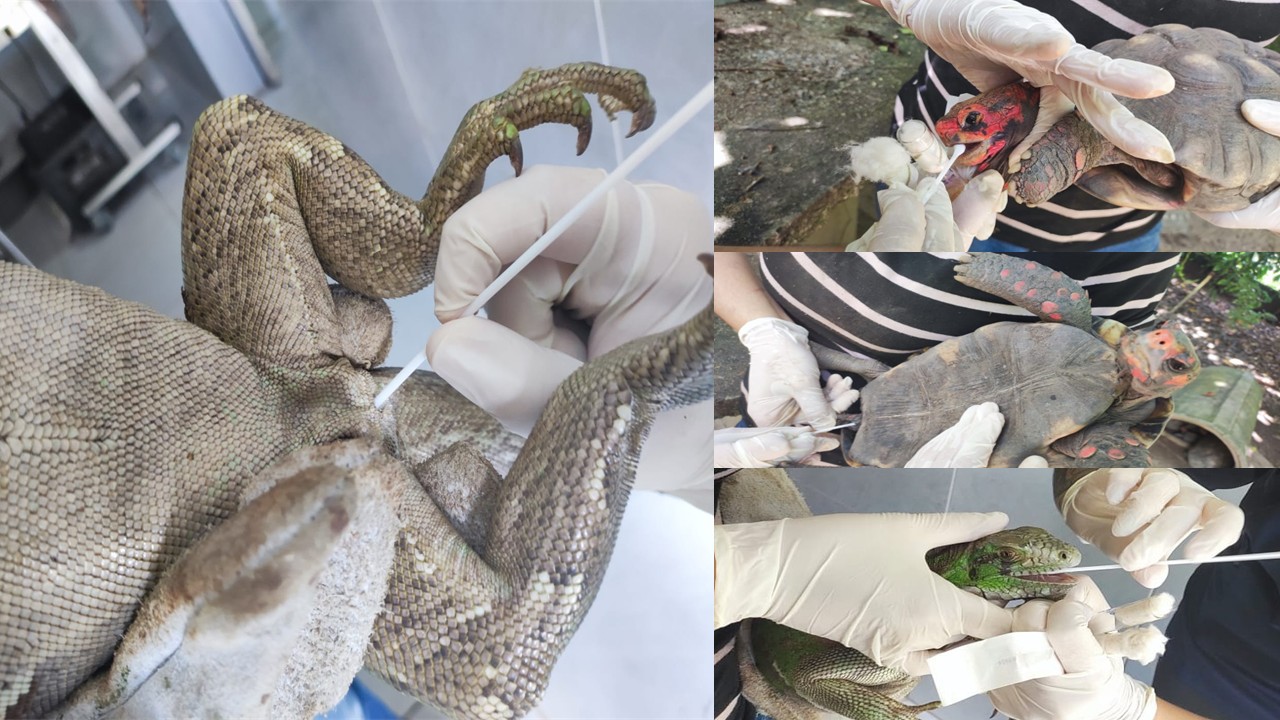

Supplement: Supplementary file 2 — Supplementary Material 2 (DOCX 249 KB) [file 42770_2026_1909_MOESM2_ESM.jpg]
